# A NAC Transcription Factor TuNAC69 Contributes to ANK-NLR-WRKY NLR-Mediated Stripe Rust Resistance in the Diploid Wheat *Triticum urartu*

**DOI:** 10.3390/ijms23010564

**Published:** 2022-01-05

**Authors:** Yang Xu, Shenghao Zou, Hao Zeng, Wei Wang, Bin Wang, Huan Wang, Dingzhong Tang

**Affiliations:** 1 State Key Laboratory of Ecological Control of Fujian-Taiwan Crop Pests, Key Laboratory of Ministry of Education for Genetics, Breeding and Multiple Utilization of Crops, Plant Immunity Center, Fujian Agriculture and Forestry University, Fuzhou 350002, China; xuyang@fafu.edu.cn (Y.X.); shenghao_zou@126.com (S.Z.); biozengh@163.com (H.Z.); wangwei@fafu.edu.cn (W.W.); 2 College of Life Science, Fujian Agriculture and Forestry University, Fuzhou 350002, China; wangbin_doc@163.com; 3 College of Agronomy, State Key Laboratory of Wheat and Maize Crop Science, and Center for Crop Genome Engineering, Longzi Lake Campus, Henan Agricultural University, Zhengzhou 450046, China

**Keywords:** NLR, YrU1, TuNAC69, stripe rust, plant immunity

## Abstract

Stripe rust is one of the most devastating diseases in wheat. Nucleotide-binding site (NBS) and leucine-rich repeat (LRR) domain receptors (NLRs) recognize pathogenic effectors and trigger plant immunity. We previously identified a unique NLR protein YrU1 in the diploid wheat *Triticum urartu*, which contains an N-terminal ANK domain and a C-terminal WRKY domain and confers disease resistance to stripe rust fungus *Puccinia* *striiformis* f. sp. *Tritici* (*Pst*). However, how YrU1 functions in disease resistance is not clear. In this study, through the RNA-seq analysis, we found that the expression of a NAC member *TuNAC69* was significantly up-regulated after inoculation with *Pst* in the presence of YrU1. TuNAC69 was mainly localized in the nucleus and showed transcriptional activation in yeast. Knockdown *TuNAC69* in diploid wheat *Triticum urartu* PI428309 that contains YrU1 by virus-induced gene silencing reduced the resistance to stripe rust. In addition, overexpression of *TuNAC69* in *Arabidopsis* enhanced the resistance to powdery mildew *Golovinomyces cichoracearum*. In summary, our study indicates that TuNAC69 participates in the immune response mediated by NLR protein YrU1, and likely plays an important role in disease resistance to other pathogens.

## 1. Introduction

Stripe rust, caused by *Puccinia striiformis* f. sp. *tritici* (*Pst*), is one of the most devastating diseases of crops that poses a major threat to the production of hexaploid wheat (*Triticum aestivum* L.), around the world [1,2]. To date, in the effort to fight for wheat stripe rust, several stripe rust resistance genes have been identified and cloned, including *Yr5/Yr7*, *YrSP*, *Yr15*, *Yr18/Lr34*, *Yr36*, *Yr46*, *YrAS2388* and *YrU1* [3,4,5,6,7,8]. Among these, *YrAS2388* encodes a nucleotide-binding site (NBS) and leucine-rich repeat (LRR) proteins (NLRs), and *Yr5*/*Yr7* and *YrSP* encode NLR with an additional BED domain [9]. *YrU1* encodes an NLR, with an N-terminal ankyrin-repeat and a C-terminal WRKY domain, which exists only in *Triticum* species, representing a very unique structure in plants [8]. The wheat stripe resistances mediated by those NLRs are race-specific and are generally only effective against a subset of *Pst* isolates.

Plant NLRs recognize pathogenic effectors and lead to effector-triggered immunity (ETI) [10,11]. The expression of many defense-related genes are up-regulated in ETI, indicating that transcription factors play key roles in this process. Although some reports show that plant NLRs can directly interact with transcription factors, how NLRs regulate defense responses dynamically by transcription factors is still unclear [12,13,14].

The NAC (NAM, ATAF1/2 and CUC2) transcription factor family is one of plant-specific transcription factor families [15]. The first member of the NAC transcription factor family, *PhNAM* (*No apical meristem*), was cloned from *Petunia hybrid*. *PhNAM* plays a role in the formation and differentiation of apical meristem of *Petunia hybrid* [16]. Subsequently, more members of the NAC family were cloned, such as *CUC* (*Cup-shaped cotyledon*) [17]. Some NAC members are found to play an important role in plant immunity. For instance, *Arabidopsis ATAF1* plays a negative role in disease resistance. The *Arabidopsis ataf1* mutants are more resistant to *Pseudomonas syringae* pv. *tomato* DC3000 and *Botrytis cinerea*, while *Arabidopsis* overexpression *ATAF1* plants are more susceptible to *Botrytis cinerea* [18]. The *Arabidopsis anac019 anac055* mutants display enhanced resistance to *Botrytis cinerea*, while overexpressing *ANAC019* or *ANAC055* led to reduced resistance to *Botrytis cinerea* [19,20]. In barley, transient overexpression of *HvNAC6* can improve the penetration resistance to powdery mildew fungus [21]. In wheat, *TaNACL-D1* and *TaFROG* enhanced the resistance of wheat to *Fusarium graminearum* [22]. In contrast, *TaNAC30*, *TaNAC21/22* and *TaNAC35* play a negative regulatory role in wheat resistance to stripe rust and leaf rust [23,24,25]. However, there is very little information about whether NAC transcription factors directly participate in NLR-mediated resistance in plants.

The diploid wheat species *Triticum urartu* is the progenitor species of the A genome of bread wheat. Previously, we identified a stripe rust resistant NLR protein YrU1 and a powdery mildew resistance NLR protein Pm60 in *T. urartu* accession PI428309 [8,26]. In this study, we found that the expression level of *TuNAC69* increased significantly in RNA-seq analysis in PI428309 upon stripe rust infection. Stripe rust resistance, but not powdery mildew resistance, was reduced in PI428309 by knocking down the *TuNAC69* by barley stripe mosaic virus (BSMV)-induced gene silencing (VIGS), indicating that TuNAC69 participates in the immune response mediated by YrU1. In addition, the penetration resistance of wheat to powdery mildew fungus was increased by overexpression of *TuNAC69* in single-cell assays. Furthermore, overexpression of *TuNA69* in *Arabidopsis* enhanced the resistance of powdery mildew, indicating that TuNAC69 may play an important role in disease resistance to other pathogens.

## 2. Results

### 2.1. The Transcript Levels of TuNAC69 Were Up-Regulated after Pst Infection

**Figure 1:**
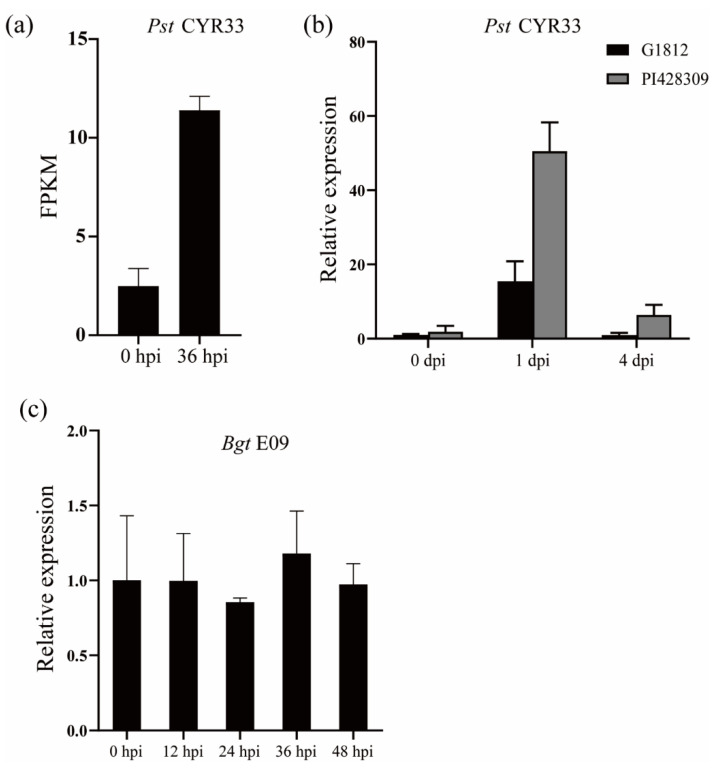
Expression analysis of *TuNAC69* in PI428309 and G1812. (**a**) RNA-seq results showed that the expression level of *TuNAC69* in PI428309 was significantly up-regulated after inoculation with *Pst*. (**b**) *TuNAC69* was up-regulated in PI428309, and was still maintained at a high level at 4 dpi. (**c**) The expression of *TuNAC69* was not significantly changed after inoculation with *Bgt* E09.

To identify genes involved in YrU1-mediated stripe resistance, we analyzed RNA-seq data in *T. urartu* accession PI428309, which contains the functional *YrU1* gene. The transcripts of *TuNAC69* in PI428309 were increased significantly after inoculation with *Pst* CYR33 (Figure 1a). To verify the results of RNA-seq, we examined the *TuNAC69* transcript levels by qRT-PCR at 0 days post-inoculation (dpi), 1 dpi and 4 dpi with *Pst* CYR33. As shown in Figure 1b, the transcripts of *TuNAC69* accumulated at a much higher level at 1 dpi and 4 dpi. We also analyzed the transcripts levels of *TuNAC69* in the susceptible *T. urartu* accession G1812, which lacks the functional *YrU1* gene. After inoculation, the transcripts of *TuNAC69* in PI428309 were much higher than that in G1812 at 1 dpi and 4 dpi. On 4 dpi, the transcript level of *TuNAC69* in G1812 was similar to the uninfected plants, while in PI428309, the transcript level was still much higher than that in the uninfected plants (Figure 1b). PI428309 also contains a functional powdery mildew resistance gene, *Pm60*, and confers resistance to *Blumeria graminis* f. sp. *tritici* (*Bgt*) [26]. In order to examine whether *TuNAC69* is induced by powdery mildew pathogen, we infected PI428309 with *Bgt* E09 and examined the transcripts of *TuNAC69* at different time points after *Bgt* E09 inoculation. In contrast, there was no significant change in the transcript levels of *TuNAC69* after inoculated with *Bgt* E09 (Figure 1c). These results indicate that *TuNAC69* may be involved in the resistance for YrU1-mediated stripe rust of wheat.

### 2.2. TuNAC69 Is Well Conserved among Plant Species

**Figure 2:**
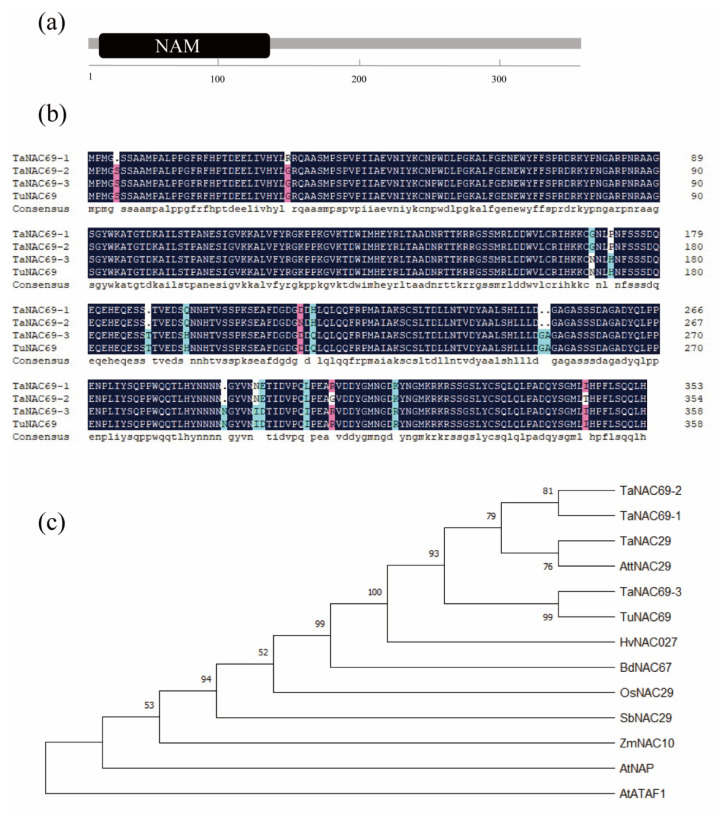
Analysis of TuNAC69. (**a**) Schematic representation of TuNAC69 protein domain. (**b**) Multiple comparisons between TuNAC69 and its homologs in wheat. (**c**) Phylogenetic analysis of TuNAC69. Different NAC transcription factors from *Triticum aestivum* (Ta), *Hordeum vulgare subsp. Vulgare* (Hv), *Aegilops tauschii subsp. tauschii* (Att), *Sorghum bicolour* (Sb), *Zea mays* (Zm), *Brachypodium distachyon* (Bd) and *Arabidopsis thaliana* (At) were used for the phylogenetic analyses. The unrooted phylogenetic tree was depicted using the MEGA X program and was constructed using the neighbor-joining method. TaNAC69-1 (AAU08785.1), TaNAC69-2 (XP_037440448.1), TaNAC69-3 (AAY44098.1), HvNAC027 (CBZ39285.1), TaNAC29 (AKC34071.1), OsNAC29 (XP_015615093.1), AttNAC29 (XP_020166974.1), AtNAP (NP_564966.1), AtATAF1 (NP_171677.1), BdNAC67 (XP_003577115.1), SbNAC29 (XP_002448919.1), ZmNAC10 (PWZ45967.1).

The full length of *TuNAC69* coding sequence encodes a protein of 359 amino acids, with a relative molecular weight of about 40 kDa. TuNAC69 has the typical structure of NAC family members, with a conserved N-terminal NAM domain (Figure 2a). In the genome of *Triticum aestivum*, *TaNAC69-1*, *TaNAC69-2* and *TaNAC69-3* are highly similar to *TuNAC69* (Figure S1), located on chromosomes 5B, 5D, and 5A, respectively. Compared with them, the TuNAC69 has an amino acid sequence identity of 96.1% with TaNAC69-1, 95.8% with TaNAC69-2, 100% with TaNAC69-3 (Figure 2b). As shown in Figure 2c, the NAC69 homologous are well conserved and presented in different plant species.

### 2.3. TuNAC69 Is Localized in the Nucleus with Transcriptional Activation Activity

**Figure 3:**
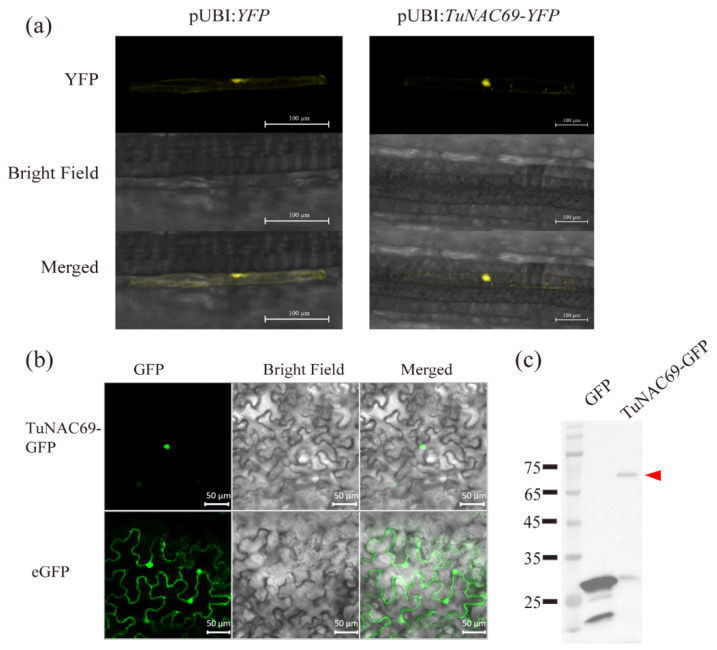
Subcellular localization of the TuNAC69-GFP fusion protein in wheat and tobacco. (**a**) TuNAC69 mainly localizes in the nucleus of *T. aestivum* cells. Bar = 100 μm. (**b**) TuNAC69 localized in the nucleus of *N. benthamiana* cells. Bar = 50. (**c**) Accumulation of TuNAC69-GFP in *N. benthamiana* leaves by immunoblot analysis. The red triangle indicates TuNAC69-GFP.

In order to examine the localization of TuNAC69, we created a construct to express TuNAC69 and Green fluorescent protein (GFP) fusion protein, then transiently expressed it in wheat and tobacco *Nicotiana benthamiana*. TuNAC69 proteins mainly localized in the nucleus in both wheat and *N. benthamiana* cells (Figure 3a,b), which is consistent with the localization characteristics of transcription factors. Immunoblot analysis indicated that TuNAC69-GFP protein was correctly expressed in *N. benthamiana* (Figure 3c).

**Figure 4:**
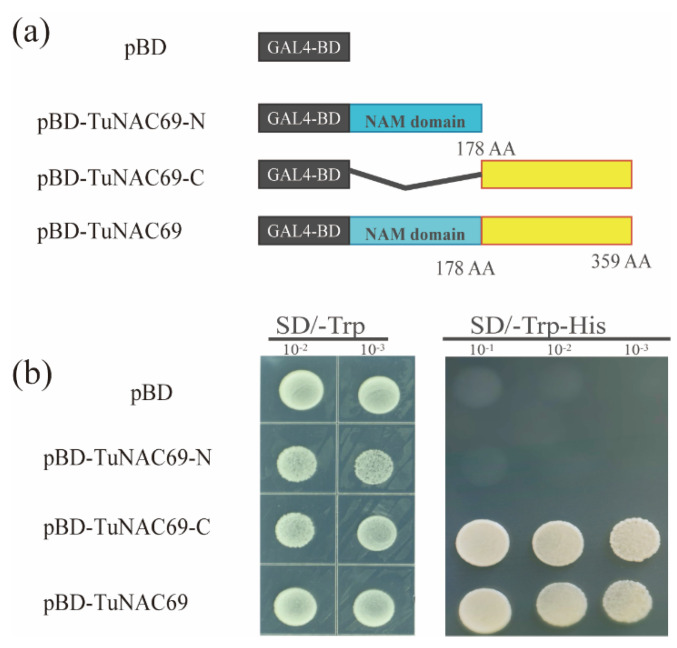
The C-terminal of TuNAC69 has transcriptional activation activity. (**a**) Schematic diagram indicated the GAL4-BD-fused TuNAC69 and truncated proteins of TuNAC69. From 1 to 178 amino acids of TuNAC69 were defined as N-terminal of TuNAC69. From 179 to 359 amino acids of TuNAC69 were defined as C-terminal of TuNAC69. (**b**) TuNAC69 and its C-terminal domain showed the activity of transcriptional activator in yeast.

TaNAC69-1 was shown to be able to inhibit *TaIAA7* and *TaSHY2* expression under drought stress [27,28,29], but the amino acid sequence comparison shows that the sequence of TuNAC69 is more similar with TaNAC69-3 in *T. aestivum*. In order to examine the transcriptional activation activity of TuNAC69, we constructed a full length of TuNAC69 and its different domains with the vector pGBKT7, and then transformed them into the yeast strain. The full length of TuNAC69, the N-terminal NAM domain, and the C- terminus were assessed respectively (Figure 4a). As shown in Figure 4b, TuNAC69 had strong transcriptional activation activity, and the C-terminal of the protein provided the activity.

### 2.4. Knockdown TuNAC69 Leads to Enhanced Susceptibility to Pst in PI428309

**Figure 5:**
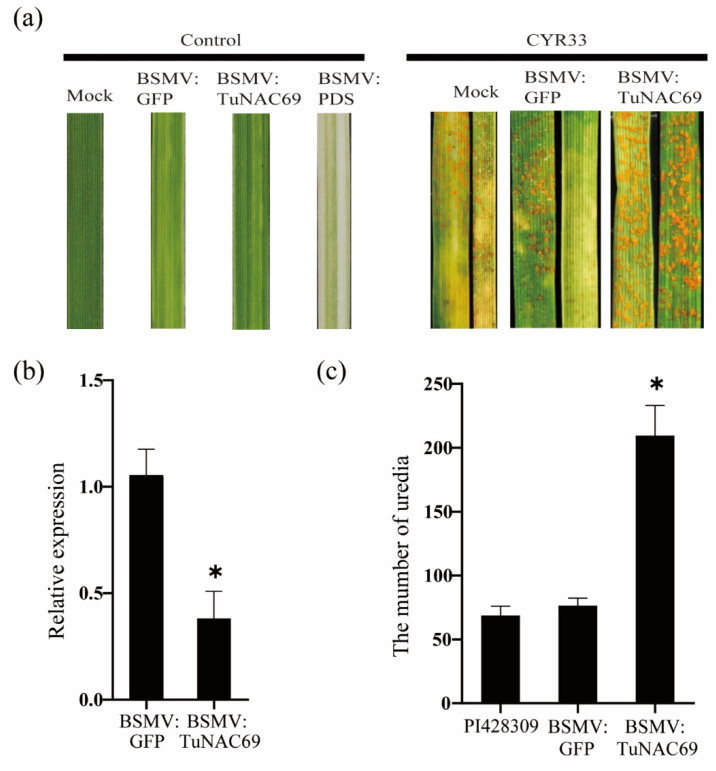
Barley stripe mosaic virus (BSMV)-induced silencing of *TuNAC69* compromised the resistance of PI428309 to *Pst* CYR33. (**a**) PI428309 was inoculated with the BSMV on the second leaf. Inoculated with urediniospores of *Pst* CYR33 on the fourth leaf after 21 days. Leaves infected with *Pst* CYR33 were examined at 14 dpi. (**b**) The transcript levels of *TuNAC69* were examined by quantitative reverse transcription PCR (qRT-PCR). *TuACTIN* was used as an internal control. (**c**) The number of uredia per cm^2^ on wheat leaves. Error bars represent ± SD of values obtained from at least three independent biological samples. Statistically significant difference (Student’s *t*-test): *, *p* < 0.05.

In order to determine whether *TuNAC69* is involved in the YrU1-mediated stripe rust resistance, we used barley stripe mosaic virus (BSMV)-induced gene silencing (VIGS) system to knockdown *TuNAC69* in PI428309, which contains a functional YrU1. Knockdown of *TuNAC69* compromised the resistance to stripe rust *Pst* CYR33. In 14 days after inoculation, the number of uredia on the leaves with reduced *TuNAC69* expression was denser than that of the control leaves, and no necrotic spot caused by cell death was observed, showing more susceptible symptoms (Figure 5).

**Figure 6:**
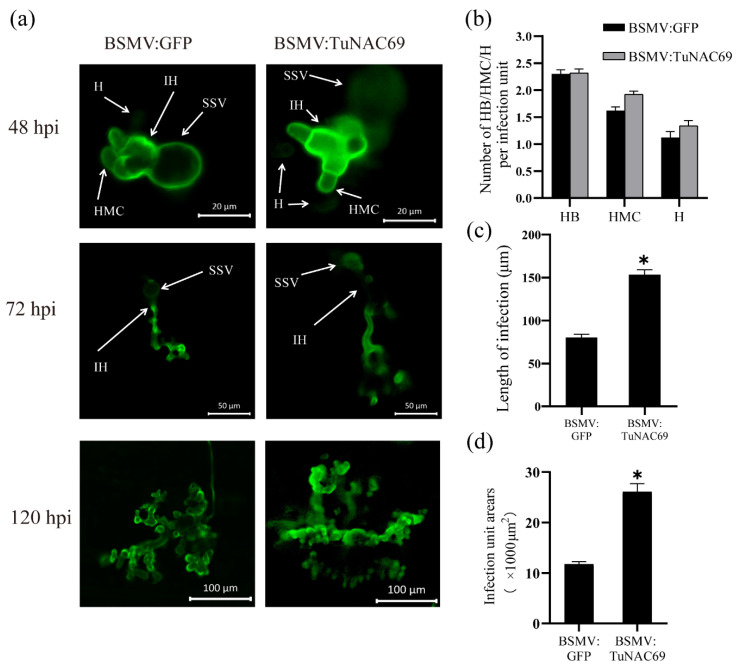
Histological observation of *Pst*. (**a**) The morphology of mycelium was observed by WGA staining at 48 hpi, 72 hpi and 120 hpi. The Bar is 20 μm, 50 μm or 100 μm, as indicated. (**b**) The numbers of HB, HMC and H of *Pst* in each infected site were counted and the average was calculated, and there was no significant difference at 48 hpi. (**c**) The length of IH in each infected site was measured at 72 hpi. (**d**) The area of each infected site of 120 hpi inoculated with stripe rust. The mycelium length and extended infection area in BSMV:*TuNAC69* plants were measured. Values represent the mean ± SE of three independent samples (*n* = 50). Statistically significant difference (Student’s *t*-test): *, *p* < 0.05.

In addition, to further characterize the stripe resistance, the infected leaves of wheat were taken at 48 hpi, 72 hpi and 120 hpi, and the mycelium of *Pst* was stained with wheat germ agglutinin (WGA), and the fungal growth was examined by confocal microscope. The number of hyphal branches (HB), haustorial mother cells (HMC) and haustoria (H) at 48 hpi were counted, respectively. The length of infection hyphae at 72 hpi infection and the infection unit area at 120 hpi were also measured [30]. As shown in Figure 6b, there was no significant difference, in the number of HB, HMC and H, between the *TuNAC69* silenced plants and the control group. However, the length of infection hyphae and the infection unit area in *TuNAC69* silenced plants were significantly larger than those in the control group (Figure 6c,d). Taken together, those results indicated that *TuNAC69* plays a positive role in the resistance of PI428309 to stripe rust.

Besides of the stripe resistance of the *YrU1* gene, PI423809 also contains a powdery mildew resistance gene *Pm60*, which is highly resistant to *Bgt* E09 [26]. In order to test whether *TuNAC69* is specifically involved in YrU1-mediated stripe rust resistance, we inoculated the *TuNAC69* silenced PI423809 plants and the control group of PI428309 with *Bgt* E09. Silencing of *TuNAC69* did not affect powdery mildew resistance (Figure S2), indicating that *TuNAC69* is not involved in Pm60-mediated resistance to *Bgt* E09 in PI428309.

### 2.5. Transient Overexpression of TuNAC69 Enhanced the Resistance to Powdery Mildew in Wheat

**Figure 7:**
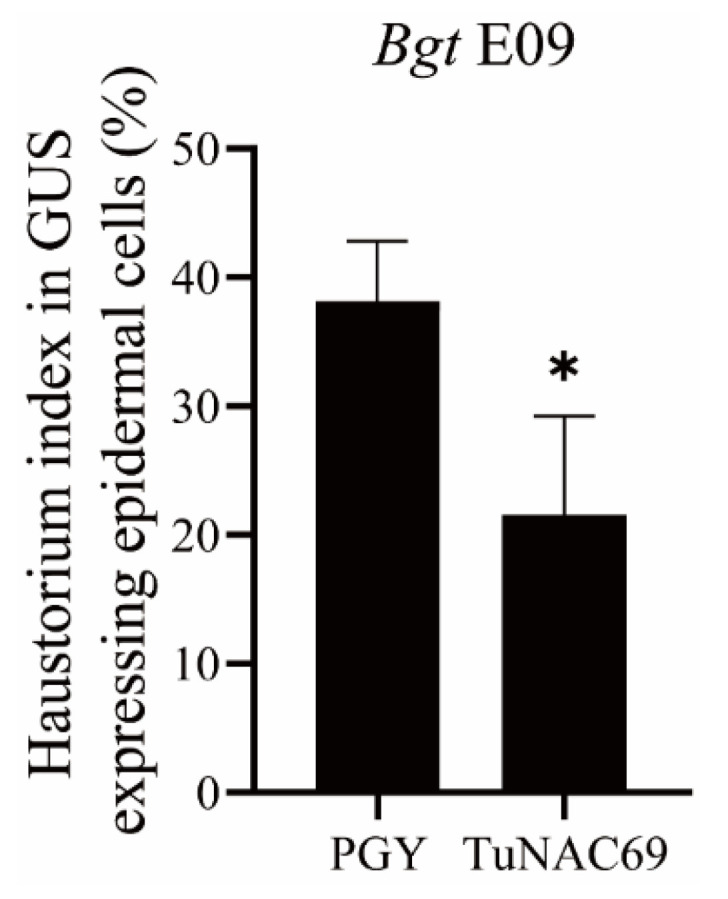
Transient overexpression of *TuNAC69* enhanced the resistance to powdery mildew in wheat. Single-cell transient expression of *TuNAC69* on the detached leaves of the susceptible wheat accession Fielder. Compared with control *PGY*, overexpression of *TuNAC69* significantly reduced haustorium index after inoculation with *Bgt* E09. Statistically significant difference (Student’s *t*-test): *, *p* < 0.05.

To study whether *TuNAC69* is involved in basal defense and disease resistance to other pathogens, we first performed transient expression mediated by particle bombardment, and then assessed the effects on the powdery mildew resistance. In this assay, we transiently expressed *TuNAC69* in the susceptible *T. aestivum* accession Fielder. As shown in Figure 7, transient expression of *TuNAC69* in the leaves of Fielder significantly decreased the haustorium index when compared to transient expression of PGY, and the empty vector used as a control, indicating that TuNAC69 plays positive roles in resistance against *Bgt* E09.

### 2.6. Overexpression of TuNAC69 Enhanced the Resistance to Powdery Mildew in Arabidopsis

**Figure 8:**
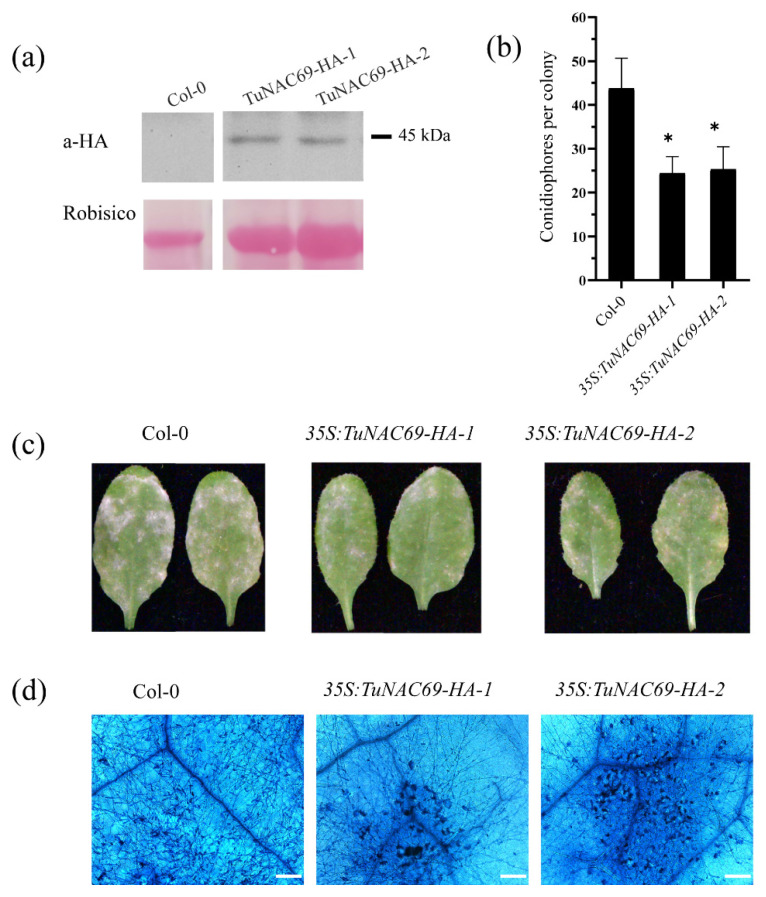
The TuNAC69-overexpressing *Arabidopsis* exhibits increased resistance to *G. cichoracearum.* (**a**) Accumulation of TuNAC69 protein was examined by immunoblot analysis in the transgenic *Arabidopsis*. (**b**) Quantitative analysis of conidiophore formation in Col-0, *35S:TuNAC69-HA-1* and *35S:TuNAC69-HA-2* plants at 5 dpi. Error bars represent ± SD from three independent experiments (*n* > 30). Statistically significant difference (Student’s *t*-test): *, *p* < 0.05. (**c**) Four-week-old plants were infected with *G. cichoracearum* and the representative leaves were photographed at 7 dpi. (**d**) Trypan blue staining of leaves after infection with *G. cichoracearum* at 7 dpi (Bar = 200 μm).

To further study the role of TuNAC69 in disease resistance, we overexpressed *TuNAC69* in the model plant *Arabidopsis thaliana*, and infected plants with powdery mildew fungus. The TuNAC69 fused with a HA tag was driven by 35S promoter, and was transformed to *Arabidopsis thaliana* accession Col-0. As shown in Figure 8a, the TuNAC69-HA protein was properly accumulated. Two independent transgenic plants overexpressing TuNAC69 (*35S:TuNAC69-HA-1*, *2*) were inoculated with powdery mildew fungus *G. cichoracearum*, and the number of conidiophores was counted at 5 dpi. As shown in Figure 8b, the conidiophores per colony in overexpressed plants were significantly lower than that of control plants (Figure 8b). In 7 dpi, the leaves of the two representative overexpressed lines supported much less growth of powdery mildew fungi, and displayed some necrotic cell death (Figure 8c,d). These results indicate that overexpression of *TuNAC69* in *Arabidopsis thaliana* can increase the resistance to *G. cichoracearum* and lead to cell death in plants.

## 3. Discussion

YrU1 is a unique NLR protein containing an ANK domain and a WRKY domain, which only exists in wheat relatives. Understanding the molecular mechanism of YrU1 is important for wheat disease resistance breeding and the interpretation of the mechanism of NLR proteins. In this study, through analyzing the RNA-seq data, we found that a NAC transcription factor *TuNAC69* was up-regulated in *Pst* CYR33 infected leaves in *T. urartu* PI428309, which contains the functional stripe rust resistance *YrU1* gene and powdery mildew resistance *Pm60* gene. The silencing of *TuNAC69* in PI428309 reduced the resistance to *Pst* CYR33, but had no effect on powdery mildew resistance to *Bgt* E09. Those results indicated that *TuNAC69* may be involved in YrU1-mediated immune response, but not Pm60-mediated resistance.

Previous works have shown that NAC transcription factors play important roles in biotic and abiotic stresses [31,32]. In wheat, the expression of *TaNAC69*, the close homolog of *TuNAC69*, was up-regulated by drought and cold [28]. Overexpression of *TaNAC69* led to enhanced expression levels of some stress up-regulated genes. And TaNAC69 and its rice homolog are able to bind to the promoter elements of three rice genes, including chitinase, ZIM, and glyoxalase I and an *Arabidopsis* glyoxalase I family gene, which are homologs of the up-regulated stress genes in the overexpression of *TaNAC69* [29]. *TaNAC69-1* overexpression in the roots (with root-predominant promoters) led to lower expression of *TaSHY2* and *TaIAA7*, the homologs of negative root growth regulators in *Arabidopsis*. Further experiments showed that TaNAC69-1 binds to *TaSHY2* and *TaIAA7* promoters as a transcriptional repressor, and is likely to function in promoting root elongation in drying soil [27]. Emerging evidence show that TaNAC69 is also involved in plant disease resistance. Analyses of the Affymetrix expression data showed that the expression of *TaNAC69* gene was up-regulated in rust-infected wheat plants [29]. RNA-seq data and quantitative real-time PCR analysis showed that the expression of *TaNAC069*, which is highly homologous to *TaNAC69*, was induced by leaf rust pathogen, and the resistance to leaf rust was significantly reduced in *TaNAC069*-silenced plants [33]. Some upstream transcription factors and interacting targets of TaNAC069 were identified. However, how *TaNAC069* contributes to leaf rust, and whether those interacting targets function in plant disease resistance are remained to be determined.

In the previous study, we reported that the ANK and WRKY domain containing NLR protein YrU1 plays important roles in wheat stripe rust resistance. Although how YrU1 functions is not clear, it has been hypothesized that the WRKY domain may bind the pathogen effector resulting in the activated of disease resistance. And the ANK domain may recruit additional components to activate downstream signaling [8]. In this study, we showed that the transcription factor TuNAC69 is required by YrU1-mediated stripe resistance. NLRs usually activate plant immunity by the perception of pathogen effectors. Whether TuNAC69 is activated after perception of pathogen effectors by YrU1 and how TuNAC69 modulates YrU1-mediated resistance are remained to be determined. Previously, it has been shown that some plant transcription factors directly interact with NLRs, and play critical roles in NLR-mediated resistance. For instance, in barley, intracellular mildew A (MLA 10) NLR protein functions in the nucleus to confer resistance against the powdery mildew fungus. Recognition of the fungal avirulence A10 effector by MLA10 induces nuclear associations between MLA10 and WRKY transcription factors HvWRKY1/2. And HvWRKY1/2 proteins act as repressors of basal defense [34]. Further study showed that barley NLR MLA10 directly interacts with two antagonistically acting transcription factors, MYB6 and WRKY1 [35]. The transcription factor MYB6 associates with MLA10 and functions as an immediate and positive signaling component for the transcriptional reprogramming to initiate disease resistance [34]. In addition, rice transcription factor OsWRKY19 interacts with NLR protein OsRLR1 and contributes to resistance to the fungal pathogen *Magnaporthe oryzae* and the bacterial *pathogen Xanthomonas oryzae* pv. *oryzae* mediated by OsRLR1 [36]. The transcription factor TuNAC69 is localized in the nucleus with transcriptional activation activity. It would be interesting to examine whether TuNAC69 interact with YrU1, and whether the interaction contributes to stripe rust resistance.

Interestingly, although transiently silenced *TuNAC69* in *T. urartu* PI428309 had no effect on Pm60-mediated powdery mildew resistance, overexpression of *TuNAC69* enhanced the resistance to powdery mildew in susceptible common wheat and *Arabidopsis*. In those cases, there are no functional NLRs present, suggesting that TuNAC69 may also function in basal defense. Based on the above results, we speculated that TuNAC69 is not only involved in YrU1-mediated immune response but also plays an important role in resistance to other pathogens. TuNAC69 is well conserved and present among plant species, and it would be very interesting to investigate whether NAC69 in other plant species also plays important roles in plant disease resistance.

Taken together, we show that TuNAC69 is localized in the nucleus with transcriptional activation activity and is involved in YrU1-mediated immune response. Our findings provide new insight into the role of YrU1 and TuNAC69 in wheat rust resistance and broaden the understanding of the role of NAC transcription factors in plant disease resistance. The important questions to be addressed include how plants transduces the immune signal to TuNAC69 to modulate transcriptional reprogramming in defense responses, and what are the direct target genes of TuNAC69, which contribute to YrU1-mediated resistance and basal immunity.

## 4. Materials and Methods

### 4.1. Plant Materials and Pathogens Growth Conditions

Plants used in this study were grown in a greenhouse at 22 °C with approximately 60% relative humidity under a 9 h-light/15 h-dark photoperiod, unless indicated otherwise. *T. urartu* accession PI428309 inoculated with *Pst* was cultured in the greenhouse at 16 °C and 16-h-light/8-h-dark photoperiod. The hexaploid wheat inoculated with *Bgt* E09 was cultured in an incubator at 22 °C under a 12 h:12 h, light:dark photoperiod. The hexaploid wheat cultivar Mingxian169 was used to maintain the *Pst* CYR33 used in the experiments. And hexaploid wheat cultivar Fielder was used to maintain the *Bgt* E09. Powdery mildew (*Golovinomyces cichoracearum* UCSC1) was cultivated with *Arabidopsis pad4-1* [37].

### 4.2. Quantitative Real-Time RT-PCR

Extraction of total RNA using TRIzol reagent, synthesis of first-strand cDNA and RT-qPCR were performed as described previously [38]. The *TuACTIN* gene was used as an internal control.

### 4.3. Phylogenetic Analyses

A full length of *TuNAC69* (*TRIUR3_14542*) was identified based on the sequence of the corresponding gene in *T. urartu* G1812. The corresponding primers were designed respectively, and the *TuNAC69* gene fragment was obtained by PCR using PI428309 cDNA as a template. The primers used in this research are listed in Table S1. The sequences of NAC transcription factors were retrieved using the NCBI database (https://www.ncbi.nlm.nih.gov/ (accessed on 13 September 2021)) and Gramene database (https://ensembl.gramene.org/index.html (accessed on 21 June 2021)). The protein domains were predicted by the NCBI SMART program (http://smart.embl-heidelberg.de/ (accessed on 23 August 2021)) and UniProt database (https://www.uniprot.org/ (accessed on 7 October 2021)). The sequences were aligned to generate a phylogenetic tree by using MEGA X.

### 4.4. Virus Induced TuNAC69 Gene Silencing

Barley stripe mosaic virus (BSMV) induced gene silencing system (VIGS) was used for functional gene verification. The 197 bp fragment of *TuNAC69* with high specificity was fused to the γ vector. The RNA α, β, γ-TuNAC69, γ-PDS and γ-GFP were generated by vitro transcription using T7 RNA polymerase. The α, β and γ-TuNAC69 (γ-PDS or γ-GFP) RNA of BSMV were mixed at equal proportion and mixed with an equal volume of GKP buffer. Then, 10 μL of the mixture was dropped and gently rubbed into the wheat leaves with a PE glove, then protected from light and moisturized for 24 h to inoculate the second fully expanded leaves of the wheat seedlings. The fifth leaves were used to evaluate the resistance to *Pst* and determine the expression levels of *TuNAC69* by qRT-PCR.

### 4.5. Histological Analysis of Fungal Growth

The leaves of different periods were decolorized with 1 M KOH with 0.05% (*v*/*v*) Silwet L-77 at 37 °C for 12 h, rinsed with 50 mM Tris (pH 7.5) for 20 min, then stained with 20 μg/mL WGA-FITC at room temperature for 15 min, and then observed with confocal microscope as described [39].

### 4.6. Single-Cell Transient Gene Expression Assay

The single-cell transient expression assay was performed as described previously [34]. The reporter plasmid containing *β-glucuronidase* (*GUS*) gene was mixed with plasmid *pUBI:PGY* or *pUBI:TuNAC69* (molar ratio 1:1; total DNA less than 2.5 μg) to express transiently in epidermal cells of wheat leaves by biolistic delivery. Leaves were incubated in GUS staining solution for about 12 h at 37 °C at 2 dpi. And then the samples were decolored and observed. The haustorium index was determined as the percentage of number of spores that developed haustorium in effective spores and the experiment was repeated three times independently, each observing at least 40 interactions.

### 4.7. Subcellular Localization Assay in Nicotiana Benthamiana

*Agrobacterium tumefaciens* GV3101 containing the recombinant plasmid *pCAMBIA1300-TuNAC69-GFP* was culture for 12 h, and collected by centrifugation at 4000 rpm and resuspended to OD_600_ = 1.5 with injection buffer (10 mM MES, 10 mM MgCl_2_, 120 µM Acetosyringone) and injected into *Nicotiana benthamiana*. Then the plants were moved to a greenhouse under a 9-h-light/15-h-dark photoperiod. GFP signals of TuNAC69-GFP were visualized by confocal microscope 48 h after infiltration.

### 4.8. Protein Extraction and Immunoblot Analysis

Plants were ground in liquid nitrogen using a mortar and pestle, total protein was extracted using the protein extraction solution (50 mM Tris-MES [pH 8.0], 0.5 M Sucrose, 1 mM MgCl_2_, 10 mM EDTA, 5 mM DTT, and 1% [*w/v*] protease inhibitor cocktail S8830) [40]. The samples were placed on ice for 30 min, turning it upside down twice, and then samples were centrifuged for 15 min at 12,000× *g* and the upper layer liquid were collected. Immunoblot analysis was performed as described previously [41].

### 4.9. Transcriptional Activation Activity of TuNAC69

Full length of *TuNAC69*, the N-terminal of *TuNAC69* (1–531 bp) and the C-terminal of *TuNAC69* (531–1077 bp) were cloned into pGBKT7, respectively. And the derived recombinant plasmids were separately transformed into *Saccharomyces cerevisiae* strain AH109. Transformed cells were cultured in SD/-Trp and SD/-Trp/-His, respectively, at 30 °C as described [42,43].

### 4.10. Genetic Transformation of Arabidopsis thaliana

The *TuNAC69* construct was introduced into the *Agrobacterium tumefaciens* strain GV3101. *Agrobacterium* with fused *TuNAC69* recombinant plasmid was suspended in a 5% (*w*/*v*) sucrose solution buffer containing 0.02% (*v*/*v*) Silwet L-77 and transformed into *Arabidopsis thaliana* accession Columbia-0 (Col-0) using the floral dip method [44]. The *Arabidopsis* were grown at a greenhouse at 22 °C under a 16-h-light/8-h-dark photoperiod for seed setting [45]. Transformants were selected on ½ Murashige and Skoog medium (½MS) containing 0.005% (*w*/*v*) kanamycin. The stably transformed plants were identified in the T3 generation and used for further analysis.

## 5. Conclusions

In this study, we found that a NAC transcription factor *TuNAC69* was up-regulated after being inoculated with *Pst* CYR33 in the presence of YrU1. TuNAC69 was mainly localized in the nucleus and showed transcriptional activation in yeast. Transiently silenced of *TuNAC69* in diploid wheat *Triticum urartu* PI42839 that contains YrU1 by VIGS reduced the resistance to stripe rust. In addition, overexpression of *TuNAC69* in common wheat and *Arabidopsis* enhanced the resistance to powdery mildew. In summary, our study indicates that TuNAC69 positively regulates YrU1-mediated stripe rust resistance and likely plays an important role in disease resistance to other pathogens.

## Data Availability

Sequence data can be found in GenBank databases under the following numbers: TaNAC69-1 (AAU08785.1), TaNAC69-2 (XP_037440448.1), TaNAC69-3 (AAY44098.1), HvNAC027 (CBZ39285.1), TaNAC29 (AKC34071.1), OsNAC29 (XP_015615093.1), AttNAC29 (XP_020166974.1), AtNAP (NP_564966.1), AtATAF1 (NP_171677.1), BdNAC67 (XP_003577115.1), SbNAC29 (XP_002448919.1), ZmNAC10 (PWZ45967.1).

## Supplementary Materials

The following supporting information can be downloaded at: https://www.mdpi.com/article/10.3390/ijms23010564/s1.
