# Supplementary material for: A NAC Transcription Factor TuNAC69 Contributes to ANK-NLR-WRKY NLR-Mediated Stripe Rust Resistance in the Diploid Wheat Triticum urartu
Source: Int J Mol Sci. 2022 Jan 5;23(1):564. doi: 10.3390/ijms23010564 (PMC8745140; doi:10.3390/ijms23010564)
Supplement: Supplementary file 1 [file ijms-23-00564-s001.zip › Figure S1.pdf]

```
TaNAC69-1   ATGCCAATGGGCAGCAGC...GCCGCCATGCCCGCCCTCCCTCCCGGCTTCCGGTTCCACCCCACCGACGAGGAGCTCATCGTCCACTAC     87
TaNAC69-2   ATGCCAATGGGCAGCAGCAGCGCCGCCATGCCCGCCCTCCCTCCCGGCTTCCGGTTCCACCCCACCGACGAGGAGCTCATCGTCCACTAC     90
TaNAC69-3   ATGCCAATGGGCAGCAGCAGCGCCGCCATGCCCGCCCTCCCTCCCGGCTTCCGGTTCCACCCCACCGACGAGGAGCTCATCGTCCACTAC     90
TuNAC69     ATGCCAATGGGCAGCAGCAGTGCCGCCATGCCCGCCCTCCCTCCCGGCTTCCGGTTCCACCCCACCGACGAGGAGCTCATCGTCCACTAC     90
Consensus   atgccaatgggcagcagc   gccgccatgcccgccctccctcccggcttccggttccaccccaccgacgaggagctcatcgtccactac

TaNAC69-1   CTCCGCAGGCAGGCCGCGTCCATGCCCAGCCCCGTGCCCATCATCGCCGAGGTCAACATCTACAAGTGCAACCCATGGGACCTCCCCGGC    177
TaNAC69-2   CTCGGCAGGCAGGCCGCGTCCATGCCCAGCCCCGTGCCCATCATCGCCGAGGTCAACATCTACAAGTGCAACCCATGGGACCTCCCCGGC    180
TaNAC69-3   CTCGGCAGGCAGGCCGCGTCCATGCCCAGCCCCGTGCCCATCATCGCCGAGGTCAACATCTACAAGTGCAACCCATGGGACCTCCCCGGC    180
TuNAC69     CTCGGCAGGCAGGCCGCGTCCATGCCCAGCCCCGTGCCCATCATCGCCGAGGTCAACATCTACAAGTGCAACCCATGGGACCTCCCCGGC    180
Consensus   ctc gcaggcaggccgcgtccatgcccagccccgtgcccatcatcgccgaggtcaacatctacaagtgcaacccatgggacctccccggc

TaNAC69-1   AAAGCTTTGTTCGGGGAGAATGAGTGGTACTTCTTCAGCCCCCGGGATCGCAAGTACCCCAACGGCGCGCGCCCGAACCGCGCCGCCGGG    267
TaNAC69-2   AAGGCCTTGTTCGGGGAGAATGAGTGGTACTTCTTCAGCCCCCGGGATCGCAAGTACCCCAACGGCGCGCGCCCCAACCGCGCCGCCGGG    270
TaNAC69-3   AAGGCCTTGTTCGGGGAGAATGAGTGGTACTTCTTCAGCCCCCGGGATCGCAAGTACCCCAACGGCGCGCGCCCCAACCGCGCCGCCGGG    270
TuNAC69     AAGGCCTTGTTCGGGGAGAATGAGTGGTACTTCTTCAGCCCCCGGGATCGCAAGTACCCCAACGGCGCGCGCCCCAACCGCGCCGCCGGG    270
Consensus   aa gc ttgttcggggagaatgagtggtacttcttcagcccccgggatcgcaagtaccccaacggcgcgcgccc aaccgcgccgccggg

TaNAC69-1   TCCGGCTACTGGAAGGCCACCGGCACCGACAAGGCCATCCTGTCCACGCCGGCCAACGAGAGCATCGGGGTCAAGAAGGCGCTCGTGTTC    357
TaNAC69-2   TCCGGCTACTGGAAGGCCACCGGCACCGACAAGGCCATCCTGTCCACGCCGGCCAACGAGAGCATCGGCGTCAAGAAGGCGCTCGTGTTC    360
TaNAC69-3   TCCGGCTACTGGAAGGCCACCGGCACCGACAAGGCCATCCTGTCCACGCCGGCCAACGAGAGCATCGGCGTCAAGAAGGCGCTCGTCTTC    360
TuNAC69     TCCGGGTACTGGAAGGCCACCGGCACCGACAAGGCCATCCTGTCCACGCCGGCCAACGAGAGCATCGGCGTTAAGAAGGCGCTCGTCTTC    360
Consensus   tccgg tactggaaggccaccggcaccgacaaggccatcctgtccacgccggccaacgagagcatcgg gt aagaaggcgctcgt ttc

TaNAC69-1   TACAGGGGCAAGCCGCCCAAGGGCGTCAAGACCGACTGGATCATGCACGAGTACCGCCTCACCGCAGCCGACAACCGGACCACCAAGCGC    447
TaNAC69-2   TACAGGGGCAAGCCGCCCAAGGGCGTCAAGACCGACTGGATCATGCACGAGTACCGCCTCACCGCAGCCGACAACCGGACCACCAAGCGC    450
TaNAC69-3   TACCGGGGCAAGCCGCCCAAGGGCGTCAAGACCGACTGGATCATGCACGAGTACCGCCTCACCGCCGCCGACAACCGGACCACCAAGCGC    450
TuNAC69     TACCGGGGCAAGCCGCCCAAGGGCGTCAAGACCGACTGGATCATGCACGAGTACCGCCTCACCGCCGCCGACAACCGGACCACCAAGCGC    450
Consensus   tac ggggcaagccgcccaagggcgtcaagaccgactggatcatgcacgagtaccgcctcaccgc gccgacaaccggaccaccaagcgc

TaNAC69-1   AGAGGATCCTCCATGAGGCTGGATGACTGGGTGCTGTGTAGGATCCACAAGAAGTGCGGCAACTTGCCCAACTTCTCCTCCTCTGACCAG    537
TaNAC69-2   AGAGGATCCTCCATGAGGCTGGATGACTGGGTGCTGTGTAGGATCCACAAGAAGTGCGGCAACTTGCCCAACTTCTCCTCCTCTGACCAG    540
TaNAC69-3   AGAGGATCCTCCATGAGGCTGGATGACTGGGTGCTGTGTAGGATCCACAAGAAGTGCAACAACTTGCACAACTTCTCCTCCTCTGACCAG    540
TuNAC69     AGAGGATCCTCCATGAGGCTGGATGACTGGGTGCTGTGTAGGATCCACAAGAAGTGCAACAACTTGCACAACTTCTCCTCCTCTGACCAG    540
Consensus   agaggatcctccatgaggctggatgactgggtgctgtgtaggatccacaagaagtgc  caacttgc caacttctcctcctctgaccag

TaNAC69-1   GAACAGGAGCATGAGCAGGAGAGCTCCACC...GTGGAGGACTCGCAGAACAACCACACCGTGTCGTCGCCCAAGTCCGAGGCCTTCGAC    624
TaNAC69-2   GAACAGGAGCATGAGCAGGAGAGCTCCACC...GTGGAGGACTCGCAGAACAACCACACCGTGTCGTCGCCCAAGTCCGAGGCCTTCGAC    627
TaNAC69-3   GAACAGGAGCACGAGCAGGAGAGCTCCACCACCGTGGAGGACTCGCACAACAACCACACCGTGTCGTCGCCCAAGTCGGAGGCCTTCGAC    630
TuNAC69     GAACAGGAGCACGAGCAGGAGAGCTCCACCACCGTGGAGGACTCGCACAACAACCACACCGTGTCGTCGCCCAAGTCGGAGGCCTTCGAC    630
Consensus   gaacaggagca gagcaggagagctccacc   gtggaggactcgca aacaaccacaccgtgtcgtcgcccaagtc gaggccttcgac

TaNAC69-1   GGCGACGGCGACGACCACCTCCAGTTGCAGCAGTTCCGCCCCATGGCGATCGCCAAGTCGTGCTCCCTCACCGACCTGCTCAACACCGTC    714
TaNAC69-2   GGCGACGGCAACGACCACCTCCAGTTGCAGCAGTTCCGCCCCATGGCGATCGCCAAGTCGTGCTCCCTCACCGACCTGCTCAACACCGTC    717
TaNAC69-3   GGCGACGGCGACGACCAGCTGCAGCTGCAGCAGTTCCGCCCCATGGCGATCGCCAAGTCGTGCTCCCTCACCGACCTGCTCAACACCGTC    720
TuNAC69     GGCGACGGCGACGACCAGCTGCAGCTGCAGCAGTTCCGCCCCATGGCGATCGCCAAGTCGTGCTCCCTCACCGACCTGCTCAACACCGTC    720
Consensus   ggcgacggc acgacca ct cag tgcagcagttccgccccatggcgatcgccaagtcgtgctccctcaccgacctgctcaacaccgtc

TaNAC69-1   GACTACGCCGCGCTCTCGCACCTCCTCCTCGACGGCGCCGG......CGCCTCGTCGTCGGACGCCGGAGCAGACTACCAGCTGCCTCCC    798
TaNAC69-2   GACTACGCCGCGCTCTCGCACCTCCTCCTCGACGGCGCCGG......CGCCTCGTCGTCGGACGCCGGAGCAGACTACCAGCTGCCGCCG    801
TaNAC69-3   GACTACGCCGCGCTCTCGCACCTCCTCCTCGACGGCGCCGGCGCCGGCGCCTCGTCGTCGGACGCCGGAGCAGACTACCAGCTGCCGCCG    810
TuNAC69     GACTACGCCGCGCTCTCGCACCTCCTCCTCGACGGCGCCGGCGCCGGCGCCTCGTCGTCGGACGCCGGAGCAGACTACCAGCTGCCGCCG    810
Consensus   gactacgccgcgctctcgcacctcctcctcgacggcgccgg      cgcctcgtcgtcggacgccggagcagactaccagctgcc cc

TaNAC69-1   GAAAACCCACTCATCTACTCGCAGCCTCCATGGCAACAAACGCTACACTATAATAACAA...CAACGGCTACGTGAACAACGAGACCATC    885
TaNAC69-2   GAAAACCCACTCATCTACTCGCAGCCTCCATGGCAACAAACGCTACACTATAATAACAA...CAACGGCTACGTGAACAACGAGACCATC    888
TaNAC69-3   GAAAACCCGCTCATCTACTCGCAGCCTCCATGGCAACAAACGCTACACTATAATAACAATAACAATGGCTACGTGAACATCGACACCATC    900
TuNAC69     GAAAACCCGCTCATCTACTCGCAGCCTCCATGGCAACAAACGCTACACTATAATAACAATAACAACGGCTACGTGAACATCGACACCATC    900
Consensus   gaaaaccc ctcatctactcgcagcctccatggcaacaaacgctacactataataacaa   caa ggctacgtgaaca cga accatc

TaNAC69-1   GACGTGCCTCAGCTACCCGAGGCGCGCGTAGATGACTACGGCATGAATGGCGATAAGTATAACGGCATGAAGAGGAAGAGATCCAGCGGC    975
TaNAC69-2   GACGTGCCTCAGCTACCCGAGGCGGGGGTAGATGACTACGGCATGAATGGCGATAAGTATAACGGCATGAAAAGGAAAAGATCCAGCGGC    978
TaNAC69-3   GACGTGCCTCAGATACCCGAGGCCCGTGTAGATGACTACGGCATGAATGGCGATAGGTACAACGGCATGAAGAGGAAGAGGTCCAGCGGC    990
TuNAC69     GACGTGCCTCAGATACCCGAGGCCCGTGTAGATGACTACGGCATGAATGGCGATAGGTACAACGGCATGAAGAGGAAGAGGTCCAGCGGC    990
Consensus   gacgtgcctcag tacccgaggc  g gtagatgactacggcatgaatggcgata gta aacggcatgaa aggaa ag tccagcggc

TaNAC69-1   AGCTTGTACTGCAGCCAGCTGCAGCTCCCAGCGGATCAGTACAGCGGCATGCTGATCCATCCGTTCCTCAGCCAGCAGCTGCACATGTG    1064
TaNAC69-2   AGCTTGTACTGCAGCCAGCTGCAGCTCCCAGCGGATCAGTACAGCGGCATGCTGACCCATCCGTTCCTCAGCCAGCAGCTGCACATGTG    1067
TaNAC69-3   AGTTTGTACTGCAGCCAGCTGCAGCTCCCGGCGGATCAGTACAGCGGCATGCTGATCCATCCGTTCCTCAGCCAGCAGCTGCACATGTG    1079
TuNAC69     AGCTTGTACTGCAGCCAGCTGCAGCTCCCGGCGGATCAGTACAGCGGCATGCTGATCCATCCGTTCCTCAGCCAGCAGCTGCACATGTG    1079
Consensus   ag ttgtactgcagccagctgcagctccc gcggatcagtacagcggcatgctga ccatccgttcctcagccagcagctgcacatgtg
```

Figure S1. Multiple comparisons between TuNAC69 and its homologs in wheat.
