# Supplementary material for: A NAC Transcription Factor TuNAC69 Contributes to ANK-NLR-WRKY NLR-Mediated Stripe Rust Resistance in the Diploid Wheat Triticum urartu
Source: Int J Mol Sci. 2022 Jan 5;23(1):564. doi: 10.3390/ijms23010564 (PMC8745140; doi:10.3390/ijms23010564)
Supplement: Supplementary file 1 [file ijms-23-00564-s001.zip › Figure S2.pdf]

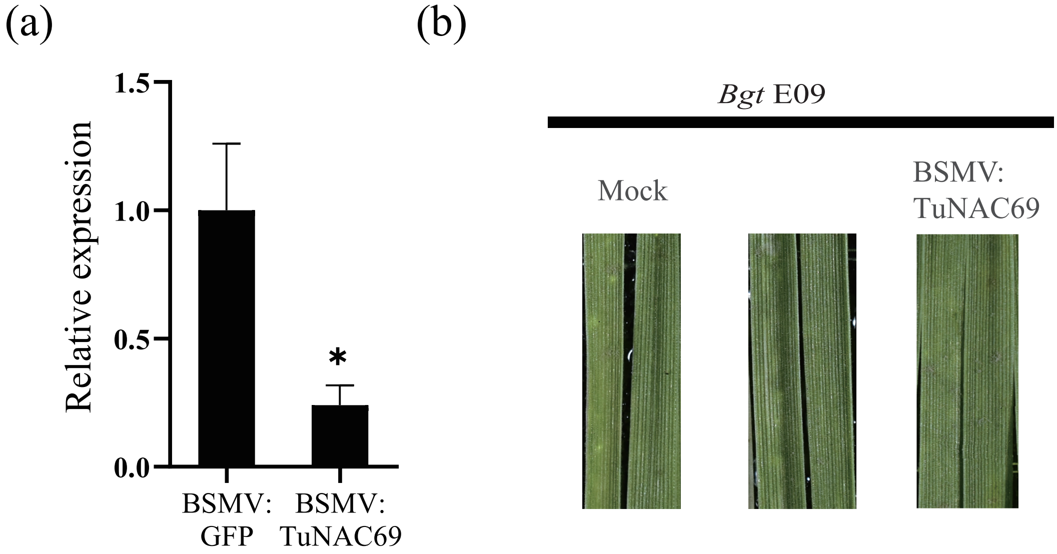

Figure S2. Knock down of *TuNAC69* by BSMV-induced gene silencing has no significant effect the resistance of PI428309 to *Bgt* E09.

(a) Analysis of VIGS silencing efficiency by qRT-PCR. *TuACTIN* was used as an internal control.

(b) PI428309 was inoculated with the BSMV on the second leaf. The fourth leaf of PI428309 was inoculated with urediniospores of *Bgt* E09 after 21 days. Leaves infected with *Bgt* were examined at 7 dpi. There was no significant difference between BSMV:GFP and BSMV:TuNAC69.
