# Supplementary material for: A NAC Transcription Factor TuNAC69 Contributes to ANK-NLR-WRKY NLR-Mediated Stripe Rust Resistance in the Diploid Wheat Triticum urartu
Source: Int J Mol Sci. 2022 Jan 5;23(1):564. doi: 10.3390/ijms23010564 (PMC8745140; doi:10.3390/ijms23010564)
Supplement: Supplementary file 1 [file ijms-23-00564-s001.zip › Figure S3.pdf]

GFP TuNAC69-GFP

anti-GFP

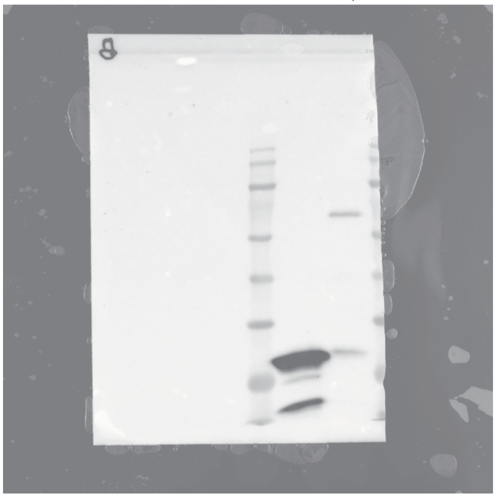

Col-0 TuNAC69-HA-1 TuNAC69-HA-2

anti-HA

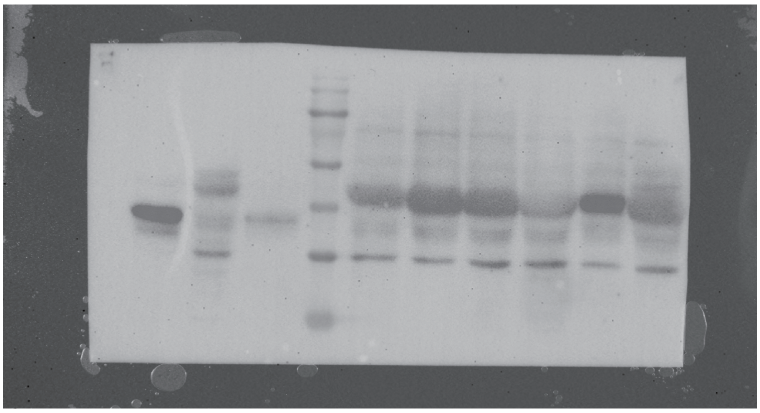

Figure S 3 . The full - size western blots gels of Figure 3c and Figure 8a.

(a) The full size western blots gels of Figure 3c

(b) The full size western blots gels of Figure 8a
